# Pregnancy Outcomes after Exposure to Tuberculosis Treatment in Phase 3 Clinical Trial, 2016–2020

**DOI:** 10.3201/eid3112.250492

**Published:** 2025-12

**Authors:** Ekaterina V. Kurbatova, William C. Whitworth, Kia E. Bryant, Meredith G. Dixon, Kelly E. Dooley, Nigel A. Scott, Rosanna Boyd, Nicole E. Brown, Kimberley N. Chapman Hedges, Wendy Carr, Lakshmi P. Peddareddy, Grace Muzanyi, Rodney Dawson, Ziyad Waja, Neil Martinson, Jyoti S.V. Mathad, Payam Nahid, Susan Swindells, Richard E. Chaisson, Susan E. Dorman, Patrick P.J. Phillips, AIDS Clinical Trials Group A5349,, Tuberculosis Trials Consortium Study 31

**Affiliations:** Centers for Disease Control and Prevention, Atlanta, Georgia, USA (E.V. Kurbatova, W.C. Whitworth, K.E. Bryant, M.G. Dixon, N.A. Scott, R. Boyd, N.E. Brown, K.N. Chapman Hedges, W. Carr, L.P Peddareddy); Vanderbilt University Medical Center, Nashville, Tennessee, USA (K.E. Dooley); Uganda–Case Western Reserve University Research Collaboration, Kampala, Uganda (G. Muzanyi); University of Cape Town Lung Institute, Cape Town, South Africa (R. Dawson); University of the Witwatersrand Perinatal HIV Research Unit, Johannesburg, South Africa (Z. Waja, N. Martinson); Weill Cornell Medical College, New York, New York, USA (J.S.V. Mathad); University of California Center for Tuberculosis, San Francisco, California, USA (P. Nahid, P.P.J. Phillips); University of Nebraska Medical Center, Omaha, Nebraska, USA (S. Swindells); Johns Hopkins University School of Medicine, Baltimore, Maryland, USA (R.E. Chaisson); Medical University of South Carolina, Charleston, South Carolina, USA (S.E. Dorman)

**Keywords:** tuberculosis and other mycobacteria, bacteria, pregnancy outcomes, congenital anomaly, fetal loss, drug-susceptible tuberculosis treatment, rifapentine

## Abstract

A previous study demonstrated noninferior efficacy of 4-month rifapentine/moxifloxacin regimen for tuberculosis (TB) treatment compared with the standard regimen. We analyzed pregnancy outcomes of women who became pregnant during the study. Among 740 women, 97 (13.1%) became pregnant. Of 102 pregnancies (in 97 participants), 30 (29.4%) participants were exposed to study drugs. Fetal loss was reported for 3/13 (23.1%) in the control regimen, 1/9 (11.1%) in the rifapentine/moxifloxacin regimen, and 1/8 (12.5%) in the rifapentine regimen. Among 21 live births in exposed pregnancies (7 in each arm), 1 infant with a congenital anomaly was reported in a participant on the rifapentine regimen. Among women receiving a short rifapentine/moxifloxacin regimen for tuberculosis who became pregnant, we observed no elevated rates of fetal losses or congenital anomalies.

Globally, among pregnant women in 2011, an estimated 216,000 had concurrent active tuberculosis (TB) (*1*). Untreated TB during pregnancy can cause pregnancy complications, nonobstetric maternal death, and infant death (*2*–*8*). Even if treated, TB during pregnancy poses challenges. For treatment of drug-susceptible pulmonary TB, standard treatment regimens containing isoniazid, rifampin, pyrazinamide, and ethambutol are highly efficacious in nonpregnant women, but well-controlled studies in pregnant women are lacking. More generally, data regarding safety, tolerability, and pharmacokinetics of TB drugs during pregnancy have not been collected or reported systematically, leading to inconsistencies in national and international treatment guidelines (*9*). For example, international guidelines recommend the use of pyrazinamide during pregnancy in first-line regimens for drug-susceptible TB (*10*), but US guidelines suggest evaluating the risks and benefits of prescribing pyrazinamide on a case-by-case basis (*11*). The scarcity of high-quality evidence combined with the worldwide occurrence of >200,000 annual cases of active TB disease among pregnant women highlights the need for additional research in this area to expand treatment options for mothers and protect the health of their infants (*9*). A February 2024 consensus statement strongly supports the participation of pregnant women in TB research (*12*), a statement that has been endorsed by community groups (*13*).

Tuberculosis Trials Consortium Study 31/AIDS Clinical Trials Group A5349 (S31/A5349) was a multicenter randomized controlled phase 3 noninferiority open-label trial that examined two 4-month treatment-shortening rifapentine-containing regimens compared with the standard 6-month control regimen for treatment of drug-susceptible pulmonary TB in nonpregnant participants >12 years of age (*14*). One investigational regimen contained rifapentine, moxifloxacin, and isoniazid administered for 4 months plus pyrazinamide administered during the first 2 months (rifapentine/moxifloxacin regimen). The other investigational regimen contained rifapentine plus isoniazid administered for 4 months plus pyrazinamide and ethambutol administered during the first 2 months (rifapentine regimen). The trial demonstrated that the 4-month rifapentine/moxifloxacin regimen had efficacy that was noninferior to that of the control and was safe and well-tolerated. The rifapentine regimen did not meet the noninferiority criteria for efficacy (*14*). The World Health Organization and the US Centers for Disease Control and Prevention (CDC) now recommend the 4-month rifapentine/moxifloxacin regimen for treatment of drug-susceptible TB in nonpregnant patients >12 years of age (*15*,*16*). The regimen is not recommended in pregnant women because they were not included in enrollment for the S31/A5349 study.

Although pregnant women were not eligible for enrollment in S31/A5349, some participants became pregnant during their participation in the study, some during study treatment, and some in the follow-up period. We conducted a secondary data analysis to describe pregnancy outcomes and safety among S31/A5349 study participants who became pregnant during the trial.

## Methods

### Study Design

Full details of the S31/A5349 study design, eligibility criteria, enrollment and randomization, safety monitoring, and study outcomes have been published previously (*14*,*17*). Pregnant or breastfeeding women were not eligible for enrollment because of uncertainties about the safety of rifapentine, moxifloxacin, and pyrazinamide in those groups (*14*,*17*). We required negative urine or serum pregnancy test results for all women of childbearing potential who were not surgically sterilized or who did not meet the study definition of postmenopausal at or within 7 days before screening. Participants of childbearing potential who were not surgically sterilized had to agree to practice an adequate method of contraception (barrier method or nonhormonal intrauterine device) or abstain from sexual activity that can lead to pregnancy during study treatment, regardless of the study regimen (*17*). Pregnancy tests during study follow-up were not required by the study protocol. We asked participants of childbearing potential during study visits about their last menstrual period, and a pregnancy test could be conducted at the discretion of clinician investigators.

### Analysis of Population, Procedures, and Definitions

We included in this secondary analysis all women who were randomized in S31/A5349, took >1 dose of assigned treatment, and became pregnant during participation in the trial. We defined a participant of childbearing potential as a woman 15–49 years of age (*18*).

For participants who reported pregnancy or were determined to be pregnant while receiving study therapy, including participants in the control arm, we permanently stopped their study treatment (regardless of assigned regimen) and treated their TB according to their respective national TB program or local guidelines (local standard of care). Pregnant participants continued to receive scheduled study follow-up except for study-specific chest radiographs (*14*,*17*). Those participants were followed by the site until the pregnancy outcome was known. We did not collect nonstudy TB treatment outcomes in this study.

We advised sites to make efforts to estimate a true conception date to the best of their ability and provided sites with guidance on using and prioritizing information available for the estimation of the conception date to determine if conception took place during study treatment and the fetus had been exposed to study drugs (*19*) (Appendix). We considered the participant to be exposed to a study drug during pregnancy if the estimated date of conception (EDC) was on or before the last study dose date. Each pregnancy report was reviewed in real-time by the safety officer (possessing an MD degree) at the Clinical Research Branch, Division of Tuberculosis Elimination, at CDC’s National Center for HIV, Viral Hepatitis, STD, and Tuberculosis Prevention.

Sites reported pregnancies on an adverse event (AE) case report form. The form included an EDC as the adverse event onset date. We captured pregnancy outcomes on an AE follow-up case report form and included live birth, fetal death (pregnancy loss at >20 weeks of gestation), spontaneous abortion (pregnancy loss at <20 weeks of gestation), or elective abortion. We defined adverse pregnancy outcomes as fetal loss (fetal death or spontaneous abortion) or infants with a congenital anomaly.

The primary efficacy outcome in the parent trial was TB disease-free survival 12 months after randomization. For each participant, we assigned a primary efficacy outcome status of favorable, unfavorable, or not assessable, as described previously; we further classified unfavorable outcomes as TB-related or not TB-related (*14*,*17*). We considered participants with unfavorable and not assessable outcomes to have a not favorable outcome.

The primary safety outcome in the parent trial was the proportion of participants with grade >3 AEs during treatment (with onset up to 14 days after the last dose of study medication). Severity of AEs was graded by the site investigators according to the National Cancer Institute common terminology criteria for adverse events version 4.03 (*20*), which requires classification of pregnancy as an AE with a grade of >3; therefore, every participant who became pregnant had >1 AE that was grade >3. We excluded the pregnancy AEs from the analysis of safety outcomes. Tolerability was a secondary safety outcome, which we defined as premature discontinuation of the assigned regimen for any reason other than microbiologic ineligibility.

The trial was approved by CDC’s Institutional Review Board and by local ethics committees, and all participants provided written informed consent. The study data were monitored by the Data Safety Monitoring Board.

### Data Analysis

We calculated length of exposure to the study drugs as the number of days between EDC and the date of the last study dose. We report frequency of pregnancy outcomes (live birth, fetal loss [death or spontaneous abortion], or elective abortion) among pregnant participants exposed to the study drugs (exposed pregnancies) in the 2 investigational arms compared with the control arm and among participants who became pregnant after study treatment was completed (unexposed pregnancies). We calculated unadjusted risk difference for fetal loss and congenital anomaly comparing investigational regimens to control with exact 95% CIs based on the 2-sided score test. We used SAS 9.4 for those calculations (*21*). We describe study TB treatment outcomes and AEs experienced by pregnant participants.

## Results

We enrolled a total of 740 female participants in S31/A5349 during January 25, 2016–October 30, 2018 (we completed study follow-up in July 2020); 97 (13.1%) of those 740 participants became pregnant during study treatment or follow-up. Five (5%) of those 97 participants became pregnant twice during trial participation, resulting in a total of 102 pregnancies. Of 102 pregnancies, 30 (29.4%) were exposed to the study drugs (either investigational or control arms). Median age of those 30 participants was 24 years (range 18–35 years); 1 participant was living with HIV (Table 1).

**Table 1:** Baseline demographic characteristics of participants who became pregnant during a TB treatment-shortening trial, Tuberculosis Trials Consortium Study 31/AIDS Clinical Trials Group A5349, January 2016–July 2020*

Characteristic	Exposed to study drugs during pregnancy, N = 30					Unexposed to study drugs during pregnancy, N = 67			
	Control, n = 13	RPT/MOX, n = 9	RPT, n = 8	Total, N = 30		Control, n = 22	RPT/MOX, n = 24	RPT, n = 21	Total, N = 67
Age at conception, y, median (range)	24 (19–35)	24 (19–29)	24 (21–33)	24 (19–35)		28 (22–38)	25 (16–44)	23 (18–41)	26 (16–44)
Age at conception group, y									
12–17†	0	0	0	0		0	1 (4.2)	0	1 (1.5)
18–34	11 (84.6)	9 (100.0)	8 (100.0)	28 (93.3)		18 (81.8)	20 (83.3)	17 (81.0)	55 (82.1)
>35	2 (15.4)	0	0	2 (6.7)		4 (18.2)	3 (12.5)	4 (19.0)	11 (16.4)
Race‡									
Asian	0	0	1 (12.5)	1 (3.3)		2 (9.1)	1 (4.2)	0	3 (4.5)
Black or African American	12 (92.3)	7 (77.8)	5 (62.5)	24 (80.0)		18 (81.8)	19 (79.2)	18 (85.7)	55 (82.1)
White	0	0	0	0		0	2 (8.3)	0	2 (3.0)
>1 race	0	2 (22.2)	2 (25.0)	4 (13.3)		1 (4.5)	2 (8.3)	3 (14.3)	6 (9.0)
Other	1 (7.7)	0	0	1 (3.3)		0	0	0	0
Not reported	0	0	0	0		1 (4.5)	0	0	1 (1.5)
Geographic region									
Africa	9 (69.2)	7 (77.8)	5 (62.5)	21 (70.0)		12 (54.5)	16 (66.7)	17 (81.0)	45 (67.2)
Asia	0	0	1 (12.5)	1 (3.3)		2 (9.1)	1 (4.2)	0	3 (4.5)
North America	3 (23.1)	2 (22.2)	1 (12.5)	6 (20.0)		7 (31.8)	5 (20.8)	3 (14.3)	15 (22.4)
South America	1 (7.7)	0	1 (12.5)	2 (6.7)		1 (4.5)	2 (8.3)	1 (4.8)	4 (6.0)
HIV-positive§	0	1 (11.1)	0	1 (3.3)		1 (4.5)	3 (12.5)	2 (9.5)	6 (9.0)
CD4 among HIV-positive, median (IQR)	NA	511 (511–511)	NA	511 (511–511)		331 (331–331)	355 (158–678)	382 (374–389)	365 (331–389)
Cavitation on baseline chest radiograph¶									
Absent	5 (38.5)	3 (33.3)	3 (37.5)	11 (36.7)		10 (45.5)	7 (29.2)	7 (33.3)	24 (35.8)
<4 cm	6 (46.2)	3 (33.3)	3 (37.5)	12 (40.0)		7 (31.8)	7 (29.2)	9 (42.9)	23 (34.3)
>4 cm	2 (15.4)	3 (33.3)	2 (25.0)	7 (23.3)		5 (22.7)	9 (37.5)	5 (23.8)	19 (28.4)
Missing	0	0	0	0		0	1 (4.2)	0	1 (1.5)
WHO sputum smear grade									
Negative	0	0	0	0		1 (4.5)	2 (8.3)	1 (4.8)	4 (6.0)
Scanty or 1–9 AFB	2 (15.4)	2 (22.2)	2 (25.0)	6 (20.0)		5 (22.7)	2 (8.3)	5 (23.8)	12 (17.9)
1+	2 (15.4)	3 (33.3)	1 (12.5)	6 (20.0)		4 (18.2)	4 (16.7)	1 (4.8)	9 (13.4)
2+	7 (53.8)	4 (44.4)	2 (25.0)	13 (43.3)		6 (27.3)	11 (45.8)	7 (33.3)	24 (35.8)
3+	2 (15.4)	0	3 (37.5)	5 (16.7)		6 (27.3)	5 (20.8)	7 (33.3)	18 (26.9)
Participant weight, kg, median (range)	49 (42–80)	49 (43–56)	51 (43–58)	50 (42–80)		51 (42–73)	50 (40–88)	50 (40–70)	50 (40–88)
BMI, kg/m^2^, median (range)	19 (18–32)	19 (17–23)	19 (16–22)	19 (16–32)		20 (16–25)	19 (15–32)	19 (15–27)	20 (15–32)
Current smoker	2 (15.4)	0	2 (25.0)	4 (13.3)		3 (13.6)	3 (12.5)	3 (14.3)	9 (13.4)
Diabetes mellitus history	2 (15.4)	0	0	2 (6.7)		2 (9.1)	1 (4.2)	1 (4.8)	4 (6.0)
Prior episode of TB treatment	0	0	1 (12.5)	1 (3.3)		1 (4.5)	2 (8.3)	1 (4.8)	4 (6.0)

*Values are no. (%) except as indicated. Participants who had >1 pregnancy during trial are shown by exposure to the study drugs for their first pregnancy. AFB, acid-fast bacillus; ART, antiretroviral therapy; BMI, body mass index; IQR, interquartile range; MOX, moxifloxacin; NA, not applicable; RPT, rifapentine; TB, tuberculosis; WHO, World Health Organization. †Minimum age of eligibility for the trial was 12 years. No pregnancies were reported in female participants 12–15 years of age. ‡Race was self-reported by trial participants. §HIV-positive persons were required to be on efavirenz-based ART for a minimum of 30 days at the time of enrollment, or, if not on ART at enrollment, planned initiation of efavirenz-based ART before or at study week 8. ¶Cavity size refers to aggregate diameter of all cavities.

Among 30 pregnancies considered exposed, the median number of days of study drug exposure was 39 days (range 11–103 days) in the control regimen, 36 days (range 13–119 days) in the rifapentine/moxifloxacin regimen, and 39 days (range 16–114 days) in the rifapentine regimen. Outcomes of 30 exposed pregnancies were 21 (70%) live births, 5 (16.7%) fetal loss (fetal death or spontaneous abortion), and 4 (13.3%) elected abortions. Fetal loss was reported for 3/13 (23.1%) pregnancies in the control regimen, 1/9 (11.1%) pregnancies in the rifapentine/moxifloxacin regimen, and 1/8 (12.5%) pregnancies in the rifapentine regimen (unadjusted risk difference [RD] −12.0% [95% CI −43.8% to 27.7%] for rifapentine/moxifloxacin vs. control arm; unadjusted RD −10.6% [95% CI −42.7% to 29.8%] for rifapentine vs. control arm) (Table 2). Four of 5 fetal losses occurred in pregnancies of <20 weeks’ gestational age. Among 21 live births in exposed pregnancies (7 in each arm), 1 infant (overall 4.8% of live births) with a congenital anomaly was reported in the rifapentine arm (1/7 live births) (unadjusted RD 14.3% [95% CI −26.0% to 53.3%] for rifapentine vs. control). That infant had congenital musculoskeletal disorder, including clubfeet and myopathy (Table 3).

**Table 2:** Duration of study drug exposure during pregnancy and pregnancy and infant outcomes in the TB treatment-shortening trial, Tuberculosis Trials Consortium Study 31/AIDS Clinical Trials Group A5349, January 2016–July 2020*

Drug exposure and outcome	Pregnancies with exposure to study drugs, N = 30					Pregnancies without exposure to study drugs, N = 72			
	Control, n = 13	RPT/MOX, n = 9	RPT, n = 8	Total, N = 30		Control, n = 24	RPT/MOX, n = 26	RPT, n = 22	Total, N = 72
Total no. study drug doses received, median (range)	157 (27–184)	118 (54–133)	107 (81–119)	118 (27–184)		181 (146–195)	119 (55–121)	118 (36–131)	119 (36–195)
Duration of study drug exposure during pregnancy, d, median (range)	39 (11–103)	36 (13–119)	39 (16–114)	37 (11–119)		NA	NA	NA	NA
Pregnancy outcome									
Live birth, no. (%)	7 (53.8)	7 (77.8)	7 (87.5)	21 (70.0)		18 (75.0)	20 (76.9)	16 (72.7)	54 (75.0)
Fetal death, no. (%)	1 (7.7)	0	0	1 (3.3)		1 (4.2)	0	2 (9.1)	3 (4.2)
Spontaneous abortion, no. (%)	2 (15.4)	1 (11.1)	1 (12.5)	4 (13.3)		0	1 (3.8)	2 (9.1)	3 (4.2)
Elective abortion, no. (%)	3 (23.1)	1 (11.1)	0	4 (13.3)		4 (16.7)	4 (15.4)	1 (4.5)	9 (12.5)
Fetal loss by fetal death or spontaneous abortion, no. (%)	3 (23.1)	1 (11.1)	1 (12.5)	5 (16.7)		1 (4.2%)	1 (3.8)	4 (18.2)	6 (8.3)
Unadjusted risk difference† from control in % with fetal loss (95% CI)‡	Referent	−12.0 (−43.8 to 27.7)	−10.6 (−42.7 to 29.8)			Referent	−0.4 (−18.4 to 16.7)	14.0 (−5.9 to 35.4)	
Unknown, no. (%)	0	0	0	0		1 (4.2)	1 (3.8)	1 (4.5)	3 (4.2)
Infant outcomes									
Congenital anomaly, no. (% of live births)	0/7 (0.0)	0/7 (0.0)	1/7 (14.3)	1/21 (4.8)		0/18 (0.0)	1/20 (5.0)	0/16 (0.0)	1/54 (1.9)
Unadjusted risk difference§ (95% CI)‡	Referent	0	14.3 (−26.0 to 53.3)			Referent	5.0 (−13.8 to 24.2)	0	

*MOX, moxifloxacin; NA, not applicable; RPT, rifapentine; TB, tuberculosis. †Difference from control in percentage with fetal loss. Exact 95% CI based on a 2-sided score test (*21*). §Difference from control in percentage of live births with congenital anomaly.

**Table 3:** Infants with congenital anomalies born to participants who became pregnant during the TB treatment-shortening trial, Tuberculosis Trials Consortium Study 31/AIDS Clinical Trials Group A5349, January 2016–July 2020*

Case	Description of congenital anomaly	Study regimen	Mother’s age at enrollment, y	Total no. study doses mother received	Total no. study doses fetus exposed	EDC calculation method	Infant’s gestational age, wks
1	Musculoskeletal disorder, including clubfeet and myopathy	RPT	26	118	41	LMP + 14 d and ultrasound in second trimester	39
2	Umbilical hernia and right inguinal hernia	RPT/MOX	22	121	0	LMP + 14 d	30

*EDC, estimated date of conception; LMP, last menstrual period; MOX, moxifloxacin; RPT, rifapentine; TB, tuberculosis.

Of 72 pregnancies considered unexposed, fetal loss was reported in 1/24 (4.2%) pregnancies in the control regimen, 1/26 (3.8%) pregnancies in the rifapentine/moxifloxacin regimen, and 4/22 (18.2%) pregnancies in the rifapentine regimen. One infant with a congenital anomaly was reported in the rifapentine/moxifloxacin regimen (1/20 live births [5.0%]). That infant had congenital umbilical hernia and right inguinal hernia (Table 3).

Among 29 participants with exposed pregnancies in the microbiologically eligible analysis population, study treatment outcome was assigned as unfavorable for 7/13 (53.8%) participants in the control regimen, 4/9 (44.4%) participants in the rifapentine/moxifloxacin regimen, and 3/7 (42.9%) participants in the rifapentine regimen (Table 4). All 14 unfavorable outcomes in this population were in the not assessable category (all were withdrawn from study treatment because of pregnancy).

**Table 4:** TB treatment outcomes in the microbiologically eligible population of the TB treatment-shortening trial, Tuberculosis Trials Consortium Study 31/AIDS Clinical Trials Group A5349, January 2016–July 2020*

Outcome	Pregnancies with exposure to study drugs, N = 29					Pregnancies without exposure to study drugs, N = 67			
	Control, n = 13	RPT/MOX, n = 9	RPT, n = 7	Total, N = 29		Control, n = 22	RPT/MOX, n = 24	RPT, n = 21	Total, N = 67
Favorable, total†	6 (46.2)	5 (55.6)	4 (57.1)	15 (51.7)		21 (95.5)	23 (95.8)	19 (90.5)	63 (94.0)
Not favorable, total‡	7 (53.8)	4 (44.4)	3 (42.9)	14 (48.3)		1 (4.5)	1 (4.2)	2 (9.5)	4 (6.0)
Unfavorable outcome, total	0	0	0	0		1 (4.5)	0	1 (4.8)	2 (3.0)
TB-related unfavorable outcome, total	0	0	0	0		1 (4.5)	0	0	1 (1.5)
Not seen at month 12; last culture positive	0	0	0	0		1 (4.5)	0	0	1 (1.5)
Not TB-related unfavorable outcome, total	0	0	0	0		0	0	1 (4.8)	1 (1.5)
Treatment changed because of adverse event	0	0	0	0		0	0	1 (4.8)	1 (1.5)
Not assessable outcome, total	7 (53.8)	4 (44.4)	3 (42.9)	14 (48.3)		0	1 (4.2)	1 (4.8)	2 (3.0)
Not seen at month 12; last culture negative	0	0	0	0		0	0	1 (4.8)	1 (1.5)
Withdrawn from treatment because of pregnancy	7 (53.8)	4 (44.4)	3 (42.9)	14 (48.3)		0	1 (4.2)	0	1 (1.5)

*Values are no. (%). Microbiologically eligible analysis population included the subset of all enrolled participants who receive a treatment assignment who, in addition, have culture confirmation of drug-susceptible TB at study entry. MOX, moxifloxacin; RPT, rifapentine; TB, tuberculosis. †All participants with favorable outcome had culture-negative status at month 12. ‡Participants with unfavorable and not assessable outcomes were considered to have a not favorable outcome.

Of 30 participants with exposed pregnancies included in the safety analysis population, 5/30 (16.7%) experienced grade >3 AEs (excluding pregnancy itself, which was always reported as an AE) during study treatment: 2/13 (15.4%) in the control regimen, 3/9 (33.3%) in the rifapentine/moxifloxacin regimen, and 0/8 (0%) in the rifapentine regimen (Table 5; Appendix Table). No deaths occurred among participants in the study who became pregnant.

**Table 5:** Safety and tolerability among participants with pregnancies in the safety population of the tuberculosis treatment shortening trial, Tuberculosis Trials Consortium Study 31/AIDS Clinical Trials Group A5349, January 2016–July 2020*

Outcome	Pregnancies with exposure to study drugs, n = 30					Pregnancies without exposure to study drugs, N = 67			
	Control, n = 13	RPT/MOX, n = 9	RPT, n = 8	Total, N = 30		Control, n = 22	RPT/MOX, n = 24	RPT, n = 21	Total, N = 67
Primary safety outcome									
Participants with grade >3 AE during study treatment†	2 (15.4)	3 (33.3)	0	5 (16.7)		5 (22.7)	2 (8.3)	0	7 (10.4)
Secondary safety outcomes									
Participants with treatment-related grade >3 AE during study treatment†	0	1 (11.1)	0	1 (3.3)		2 (9.1)	1 (4.2)	0	3 (4.5)
Other safety outcomes									
Participants with any serious AE during study treatment†	1 (7.7)	1 (11.1)	0	2 (6.7)		3 (13.6)	1 (4.2)	0	4 (6.0)
Participants died	0	0	0	0		0	0	0	0
Participants with any AE resulting in discontinuation of study treatment	0	0	0	0		1 (4.5)	0	1 (4.8)	2 (3.0)
Participants with any grade >3 AE during 28 wks after randomization†	3 (23.1)	5 (55.6)	1 (12.5)	9 (30.0)		8 (36.4)	5 (20.8)	5 (23.8)	18 (26.9)
ALT or AST 5-fold ULN‡	0	0	0	0		1 (4.5)	0	0	1 (1.5)
ALT or AST 10-fold ULN	0	0	0	0		0	0	0	0
Serum total bilirubin 3-fold ULN§	0	0	0	0		0	0	0	0
ALT or AST 3-fold ULN plus serum total bilirubin 2-fold ULN (Hy’s law)	0	0	0	0		0	0	0	0
AEs during pregnancy¶									
Participants with grade >3 AE	1 (7.7)	2 (22.2)	0	3 (10.0)		0	0	0	0
Participants with treatment-related grade >3 AE	0	0	0	0		0	0	0	0
Participants with any AE	1 (7.7)	1 (11.1)	0	2 (6.7)		0	0	0	0
Tolerability (microbiologically eligible analysis population)									
Discontinuation of assigned treatment for any reason	7 (53.9)	4 (44.4)	3 (42.9)	14 (50.0)		1 (4.6)	1 (4.2)	2 (9.5)	4 (6.0)

*ALT, alanine aminotransferase; AST, aspartate aminotransferase; MOX, moxifloxacin; RPT, rifapentine; ULN, upper limit of normal range. †Pregnancies were excluded from this analysis (all pregnancies were reported as grade >3 AE in this trial). ‡>5-fold ULN corresponds to grade >3. §>3-fold ULN corresponds to grade >3. ¶Includes AEs (other than pregnancy) with onset date from estimated date of conception through date of pregnancy outcome.

## Discussion

This analysis examined pregnancy outcomes among women who became pregnant during participation in the S31/A5349 trial of treatment of drug-susceptible pulmonary TB. We observed no excess fetal losses among pregnant participants in the rifapentine/moxifloxacin arm compared with the control arm, although the numbers were small. We noted no infants with congenital anomalies among those considered exposed to study drugs during pregnancy in the rifapentine/moxifloxacin arm. The overall percentages of fetal loss (16.7%) and congenital anomalies (4.8% of live births) in pregnancies exposed to the study drugs we observed in this trial were similar to those estimated for the United States (19.7% for fetal loss and 3% of live births for congenital anomalies) (*22*,*23*).

Because multiple antibiotics are used concomitantly during TB treatment, isolating the effects of individual drugs on pregnancy outcomes is challenging. With regard to rifapentine use in pregnant animals, previous developmental toxicity studies in rats and rabbits suggested that rifapentine produced fetal harm and was teratogenic (*24*). This description is similar to that of rifampin, which was teratogenic in high doses in animal models (*25*); however, on the basis of extensive use and years of clinical experience, the use of rifampin to treat TB during pregnancy has benefits greater than safety concerns with respect to curing TB. Studies of rifapentine during human pregnancy and lactation have been limited. In 6 patients randomized to rifapentine for initial treatment of TB in humans and who become pregnant during this trial, no episodes of teratogenicity occurred; 2 patients had normal deliveries, 2 had first-trimester spontaneous abortions (1 patient had alcohol use disorder, and the other patient was living with HIV), 1 had an elective abortion, and 1 was lost to follow-up (*24*). In analysis evaluating safety and pregnancy outcomes among pregnant women who were inadvertently exposed to study medications in 2 Tuberculosis Trials Consortium Study latent tuberculosis treatment trials (PREVENT TB and iAdhere), evaluating 3 months of weekly rifapentine (900 mg) with isoniazid and 9 months of daily isoniazid, fetal loss or congenital anomalies were at similar rates to the general population (*26*). Among 50 women enrolled in the IMPAACT 2001 trial, designed to assess 3 months of weekly rifapentine with isoniazid for TB prevention in pregnant women during the second or third trimester, with or without HIV, no drug-related serious AEs, treatment discontinuations, or TB cases were reported, although 1 case of fetal death was related to maternal physical trauma (*27*). The DOLPHIN-Moms trial, assessing 1 month of daily isoniazid and rifapentine versus 3 months of once-weekly rifapentine and isoniazid in pregnancy, is currently enrolling (*28*).

Concerning fluoroquinolone use in pregnancy, animal studies showed delayed skeletal development in fetal rats and rabbits when exposed to moxifloxacin and toxic cartilage effects in immature dogs when exposed to temafloxacin (*9*,*29*). However, in human studies, a metaanalysis that included 5 studies on use of quinolones during the first trimester of pregnancy did not find an increased risk for major malformations, stillbirths, preterm births, or low birthweight (*30*). A small case series of pregnant women with drug-resistant TB treated with second-line drugs, including fluoroquinolones, suggested that favorable pregnancy outcomes are achievable (*31*–*34*). A systematic review and metaanalysis of outcomes of pregnancies exposed to quinolone and fluoroquinolones, involving 8 cohort and 2 case–control studies, showed no statistically significant increases in rates of major malformations for quinolone and fluoroquinolone exposures (*35*).

Our study expands the scientific literature with additional data on rifapentine, moxifloxacin, pyrazinamide, and isoniazid early in pregnancy. We found that relatively brief exposures to rifapentine and moxifloxacin early in pregnancy were not associated with adverse fetal or maternal outcomes. However, the effect of longer exposures to rifapentine and moxifloxacin and effect of exposures later in pregnancy on maternal and fetal outcomes remains unknown, given that the trial specified immediate study treatment discontinuation and transition to local standard of care when a pregnancy was recognized. Our findings can help support and accelerate the participation of pregnant women in TB drug trials. In addition, publication of the detailed methodology used in this study to estimate the date of conception and total period of drug exposure during pregnancy might inform other clinical trials and thus help develop much needed harmonized reporting of safety outcomes among pregnant trial participants.

One limitation of our study is that the number of participants who became pregnant during study treatment was small, probably because of rigorous education of study participants of childbearing potential about contraception and preventing pregnancy during study treatment. The small number of pregnant participants limited the ability to detect rare events that can only be detected in larger trials and through pharmacovigilance efforts in the future. In addition, in participants who became pregnant, durations of exposures to study drugs were short, given that the protocol required immediate study treatment discontinuation in pregnancy. Adverse pregnancy events that require a higher cumulative exposure to study drugs to occur might not have been observed, so results should be interpreted in light of the short exposures. However, an average of 30 days of drug exposure during first trimester is a relatively long period of drug exposure and should not be dismissed. Furthermore, because fetal organogenesis primarily occurs in the first trimester, it is reassuring that only 1 infant with a congenital anomaly was observed among pregnancies exposed to rifapentine. In addition, because the study protocol required permanent discontinuation of the study treatment if a participant became pregnant (and transition to local standard of care), most participants who became pregnant during study treatment had their primary study TB treatment efficacy outcome classified as not assessable. Although final pregnancy outcomes were collected in the database for all participants, final TB treatment outcomes (after permanent stop of study treatment and switching to the local standard of care regimen) were not. Moreover, the study did not assess congenital anomalies among fetal deaths or spontaneous abortions. Finally, because study arms contained multiple study drugs, we cannot determine the effect of any single drug on adverse pregnancy outcomes. However, anti-TB drugs are commonly used in pregnancy, given the urgency of providing swift, full treatment for TB as soon as it is diagnosed.

In conclusion, among exposed pregnancies in this large phase 3 drug-susceptible pulmonary TB treatment trial, we did not observe a higher risk for fetal loss or infants with congenital anomalies among those participants who became pregnant while receiving a rifapentine/moxifloxacin regimen compared with the standard 6-month regimen. Those data can be used by clinicians and patients as they engage in shared decision-making and weigh the risks and benefits of using a shorter-duration 4-month rifapentine/moxifloxacin regimen versus a 6-month standard-of-care regimen in pregnancy, especially in the circumstance where an on-treatment pregnancy occurs. Future trials of rifapentine/moxifloxacin-containing regimens should consider allowing reconsent for participants who become pregnant during study treatment so that they can continue these study drugs with careful follow-up if there is potential benefit and there are no contraindications (*36*). The data from our study contribute to the growing body of information about the safety of rifapentine-containing regimens in pregnancy, which should support fuller participation of pregnant women in future TB clinical trials that include these antibiotics.

Appendix Additional information about pregnancy outcomes after exposure to tuberculosis treatment in phase 3 clinical trial, 2016–2020.
