# Supplementary material for: Pregnancy Outcomes after Exposure to Tuberculosis Treatment in Phase 3 Clinical Trial, 2016–2020
Source: Emerg Infect Dis. 2025 Dec;31(12):2233–42. doi: 10.3201/eid3112.250492 (PMC12782182; doi:10.3201/eid3112.250492)
Supplement: Appendix — Additional information about pregnancy outcomes after exposure to tuberculosis treatment in phase 3 clinical trial, 2016–2020. [file 25-0492-Techapp-s1.pdf]

Article DOI: https://doi.org/10.3201/eid3111.250492

EID cannot ensure accessibility for supplementary materials supplied by authors. Readers who have difficulty accessing supplementary content should contact the authors for assistance.

Pregnancy Outcomes after Exposure to Tuberculosis Treatment in Phase 3 Clinical Trial, 2016–2020

Appendix

Definitions and methods

Definitions and methods for estimating date of conception (EDC) in participants who became pregnant during the tuberculosis treatment shortening trial (TBTC Study 31/ACTG A5349)

1. Definitions of terms

a. Estimated date of conception (EDC) refers to the date when conception most likely occurred.

b. Gestational age (GA) refers to the length of pregnancy after the first day of the last menstrual period (LMP) and is usually expressed in weeks and days. This is also known as menstrual age.

c. Conceptional age (CA) is the true fetal age and refers to the length of pregnancy from the time of conception.

d. Estimated date of delivery (EDD), or estimated due date refers to the date when birth is expected.

e. Date of birth (DOB) refers to the actual date the infant was born.

2. Estimating the date of conception

Sites are advised to estimate a true conception date to the best of ability as it is important to determine if fetus was exposed to study drugs.

Per Manual of Operating Procedures (MOOP): “If the adverse event (AE) is a pregnancy, the onset date should be reported as the first day of last menstrual period plus 14 days.”

If last menstrual period (LMP) is not available, one or a combination of the methods described below may be used to estimate gestational age (GA) and estimated date of conception (EDC) (*EDC = date when GA was estimated – (GA (in weeks)*7) + 14 days).*

(1) Ultrasound measurement of the embryo or fetus in the first trimester up to and including 13 6/7 weeks of gestation is the most accurate method to estimate GA. Ultrasound measurements in second trimester are reasonably accurate between 14 and 22 weeks of pregnancy. Please consider obtaining an obstetrical ultrasound for pregnancies suspected to be in first and second trimester based on LMP.

(2) The actual date of conception may be reported by the participant.

(3) Date of positive pregnancy test if sensitivity of the test is known (number of days after conception when test becomes positive), (*EDC = date of the first positive pregnancy test – test sensitivity*).

In some cases, more precise estimation of EDC is only possible after pregnancy outcome is known. In such a case, it is recommended to report onset date using the best estimate available at the time of the form completion and update AE form after more precise estimate is available. Depending on what information is available after pregnancy outcome is known, calculate EDC using these formulas:

a. Estimated delivery date – 266 days

b. Date of infant’s birth – 266 days (if full term)

c. Date of infant’s birth – (GA (in weeks)*7) + 14 days (if preterm or if exact GA is known)

d. Date of abortion – (GA (in weeks)*7) + 14 days

Make a record of the method used to estimate EDC in the comments to AE and AF case report forms to the best of your ability: LMP, date and method of first positive pregnancy test, date of obstetrical ultrasound and estimated gestational age, pregnancy outcome and its date

Appendix Table. Primary safety outcome: grade 3 or higher adverse events during treatment (+14 d) by MedDRA preferred term among participants with pregnancies* in the safety population of the tuberculosis treatment shortening trial, Tuberculosis Trials Consortium Study 31/AIDS Clinical Trials Group A5349, January 2016–July 2020†

	Pregnancies with exposure to study drugs, N = 30				Pregnancies without exposure to study drugs, N = 67			
MedDRA preferred term	Control, n = 13	RPT/MOX, n = 9	RPT, n = 8	Total, N = 30	Control, n = 22	RPT/MOX, n = 24	RPT, n = 21	Total, N = 67
Grade >3 AE during study treatment, excluding pregnancies								
Brief psychotic disorder, with postpartum onset	1	0	0	1	0	0	0	0
Complication of pregnancy	0	1	0	1	0	0	0	0
Congenital anomaly	0	0	1	1	0	0	0	0
Conjunctivitis	0	1	0	1	1	0	0	1
Hepatitis	0	0	1	1	2	0	0	2
HIV infection	0	0	0	0	1	0	0	1
Hypertension	1	0	0	1	0	0	0	0
Hypochromic anemia	1	0	0	1	0	0	0	0
Malaria	0	0	0	0	0	1	0	1
Neutropenia	0	1	0	1	0	1	0	1
Overdose	0	0	0	0	1	0	0	1
Pneumonia bacterial	0	0	0	0	0	1	0	1
Pre-eclampsia	1	0	0	1	0	0	0	0
Sacroiliitis	0	0	0	0	1	0	0	1
Seizure	0	0	0	0	1	0	0	1
Tonsillitis	0	1	0	1	0	0	0	0
Upper respiratory tract infection	1	0	0	1	0	0	0	0
Urinary tract infection	1	0	0	1	1	0	0	1
Vulvovaginal candidiasis	2	1	0	3	1	0	0	1
Vulvovaginal inflammation	0	1	0	1	0	0	0	0
Total AEs	8	6	2	16	9	3	0	12
Participants with any grade >3 AE	2 (15.4)	3 (33.3)	0 (0.0)	5 (16.7)	5 (22.7)	2 (8.3)	0 (0.0)	7 (10.4)
Grade >3 AE during pregnancy‡								
Brief psychotic disorder, with postpartum onset	1	0	0	1	0	0	0	0
Complication of pregnancy	0	1	0	1	0	0	0	0
Tonsillitis	0	1	0	1	0	0	0	0
Vulvovaginal candidiasis	0	1	0	1	0	0	0	0
Total AEs	1	3	0	4	0	0	0	0
Participants with any grade >3 AE during pregnancy	1 (7.7)	2 (22.2)	0 (0.0)	3 (10.0)	0 (0.0)	0 (0.0)	0 (0.0)	0 (0.0)

*Pregnancies were excluded from this analysis (all pregnancies were reported as grade >3 AEs in this trial).
†The safety analysis population included all participants who underwent randomization and received at least one dose of the assigned treatment and became pregnant during the study. Safety was assessed during the on-treatment period (the time during which the participants were receiving the study treatment and up to 14 d after the last dose), unless otherwise specified. Adverse events were graded by the site investigators according to the National Cancer Institute Common Terminology Criteria (CTCAE) version 4.03 for Adverse Events. Some participants had more than one grade >3 AE. AE, adverse event; MedDRA, Medical Dictionary for Regulatory Activities; MOX, moxifloxacin; RPT, rifapentine.
‡Includes AEs (other than pregnancy) that had an onset date between EDC (estimated conception date = pregnancy AE onset date) and the date of pregnancy outcome (i.e., any AEs that occurred during pregnancy).
